# An Interesting Unknown Combined Pathology in a Patient with Acute Balance Problem

**DOI:** 10.1155/2019/6040852

**Published:** 2019-08-28

**Authors:** Sertac Yetiser, Kutlay Karaman

**Affiliations:** ^1^Clinical Professor, Anadolu Medical Center, Dept of ORL & HNS, Kocaeli 41400, Turkey; ^2^Clinical Fellow, Anadolu Medical Center, Dept of Radiology, Kocaeli 41400, Turkey

## Abstract

A 54-year-old woman with acute-onset nausea and vomiting presented to outpatient clinic. She had headache for 3 weeks. She had difficulty during tandem gait and was falling to the right. Otherwise, her neurological examination was normal. She had normal hearing. VNG analysis revealed spontaneous nystagmus beating to the left with optical fixation. However, she had horizontal and slightly down-beating gaze-evoked nystagmus at primary gaze position. Temporal bone CT and MRI showed widespread encephalitis of the right side of the brain and isolated destruction of the right superior semicircular canal. The patient was treated with high-dose combined antibiotics. She had remarkable recovery within 3 weeks.

## 1. Introduction

Analysis of eye movement abnormalities in patients with acute-onset balance disorders is an ideal method to investigate the function of the network controlling eye movements and presents solid evidence for radiological evaluation if necessary. True diagnosis of a vestibular pathology is always critical especially in an emergency condition. It is also a challenge if the pathology involves both peripheral and central vestibular structures [1, 2]. We present an interesting case with contralateral-beating spontaneous nystagmus with optical fixation and bilateral gaze-evoked nystagmus due to simultaneous presence of intracranial and isolated labyrinthine lesions.

## 2. Case Report

**Figure 1:**
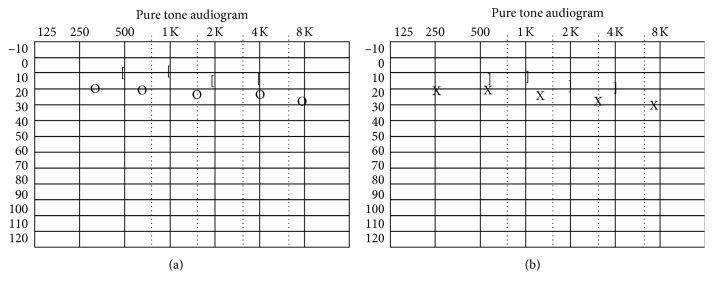
Hearing of the patient was normal as seen on pure tone audiometry. (a) Right ear. (b) Left ear.

**Figure 2:**
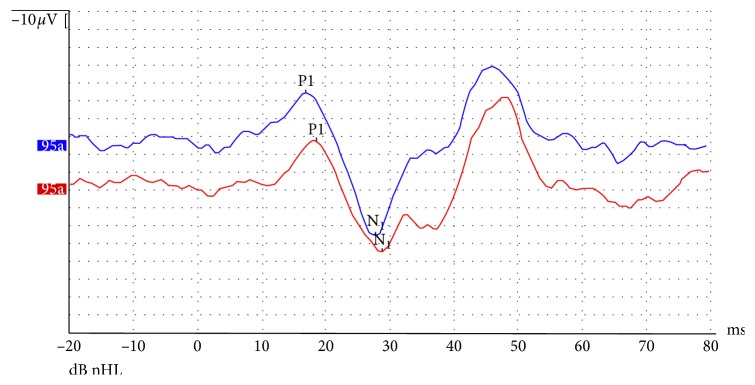
Vestibular evoked myogenic potentials (cervical) following tone-burst sound stimuli of 95 dB nHL with rarefaction polarity, 5 Hz stimulus repetition rate, 1 ms rise/fall time, and 2 ms plateau time delivered at 500 Hz. Normal waveforms were seen on both sides. But, the P_1_N_1_ amplitude decreased on the right side (red drawing, recording from RE; blue drawing, recording from LE). Comparative analysis of the amplitude of the right and left side was less than 20%.

**Figure 3:**
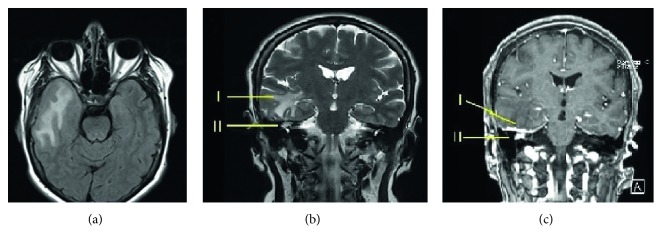
Magnetic resonance imaging of the temporal bone. (a) Axial T2 (T2-TIRM) fluid-attenuated view to suppress cerebrospinal fluid shows extensive edema of the right temporal lobe. (b) Coronal T2 image shows intracranial inflammation (I) and soft tissue located supralabyrinthine area of the right inner ear (II). (c) Coronal T1 view with gadolinium contrast shows dural thickening with contrast enhancement throughout the right tegmen (I). Soft tissue with contrast enhancement is also seen at the supralabyrinthine area (II).

**Figure 4:**
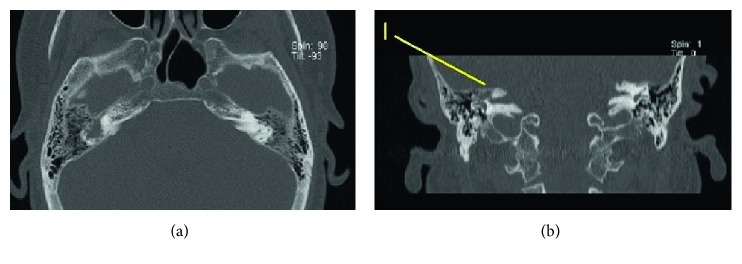
Computerized tomography of the temporal bone. (a) Axial cut crossing the supralabyrinthine area shows soft tissue filling the supralabyrinthine air cells. (b) Coronal view shows isolated destruction of the superior semicircular canal (marked with I). Normal aeration of the mastoid air cells is seen.

A 54-year-old woman with acute-onset nausea and vomiting presented to outpatient clinic. She had headache for 3 weeks. Past medical story revealed that she had been treated for sinusitis one year ago. Her quick blood work demonstrated high sedimentation only. She had difficulty during tandem gait and tended to fall to the right. Otherwise, her neurological examination (pupils, fundoscopy, search of any pathological reflex, search of neck stiffness, skew deviation, mental status, coordination, muscle strength, and examination of other cranial nerves) was normal. She had normal hearing (Figure 1). She was subjected to videonystagmographic (VNG) analysis which revealed spontaneous nystagmus beating to the left (MicroMed, Inc., Chatham, IL, USA). However, optical suppression was documented following presentation of visual target. Positional tests were normal. Smooth pursuit, optokinetic test, and sinusoidal tracking were abnormal. Vestibular evoked myogenic potentials (VEMPs) were depressed on the right side. But, comparison of both sides was within normal limits (Eclipse EP25, Interacoustics, USA) (Figure 2). She was unable to overcome the video head impulse test (VHIT). However, she had horizontal and slightly down-beating gaze-evoked nystagmus while looking to the right and left (video; gaze to the right: torsional nystagmus (upper pole to the right), with downward vertical component and gaze to the left: torsional nystagmus (upper pole to the left), with downward vertical component ([Supplementary-material supplementary-material-1])). Magnetic resonance imaging (MRI) at T2 showed hyperintense widespread inflammatory gliosis of the right temporal lobe of the brain, and temporal bone computerized tomography (CT) showed isolated destruction of the right superior semicircular canal (Figures 3(a)–3(c), 4(a), and 4(b)). Inflammatory edema was extending deeply along the right side of the brain anterior to the cerebellum as seen on MRI. Linear dural contrast enhancement adjacent to the temporal lobe was seen throughout the right tegmen. Cerebrospinal fluid (CSF) sample analysis for infection including tuberculosis was negative. However, the patient was hospitalized and treated with combined antibiotics. Caloric testing 3 days after hospitalization showed slightly decreased response on the right, but overall analysis was within normal limits. She had remarkable recovery within 3 weeks. Her VHIT was normal 3 weeks after treatment.

## 3. Discussion

Spontaneous nystagmus is the only initial sign in most patients with acute balance problem. There are usually no or very mild distinctive clinical neurological symptoms indicative of central pathology in acute-onset condition. Direction of the nystagmus could be horizontal or mostly vertical, and the intensity is unaffected by visual fixation [3]. Patients usually walk in the emergency room at the acute period without any particular functional loss. Therefore, detection of eye movement abnormalities has utmost importance in such instances [4]. On the contrary, simultaneous presentation of central and peripheral vestibular disorders is associated with overlapping signs and often time challenging. Our patient had normal hearing, and the spontaneous nystagmus at primary gaze position was beating contralateral to the lesion side. Spontaneous nystagmus seems to be due to an isolated labyrinthine dysfunction limited to the superior semicircular destruction since the nystagmus disappeared with optical stimulation. On the contrary, increase in the intensity of nystagmus with sound stimulation would confirm the peripheral pattern of spontaneous nystagmus. However, this was not evident in the presented case [5]. It was also interesting to note that the vestibular response to caloric stimulation on the lesion side was normal indicating normal functioning lateral canal. Video head impulse test in the plain of the canal would give more information for each canal function.

The patient had impaired smooth pursuit, abnormal optokinetic response, and gaze-evoked nystagmus. It has been reported that these findings are the signs for central involvement in 92% of cases [1, 2]. Vestibulocerebellar fibers carry information related to eye movements. The cerebellum transmits impulses to the brainstem neural integrator, which is a neural network located largely in the medulla for horizontal movements and in the midbrain for vertical movements. Neural integrator takes velocity commands from the conjugate eye movement systems and creates a position command to hold eyes steady after every movement. Gaze-evoked nystagmus is mainly clear manifestation of cerebellar dysfunction which indicates defective gaze-holding mechanism in the floccular region, the vestibular nucleus prepositus complex, and its connecting pathways [6, 7]. This type of nystagmus is also caused by brainstem pathology, but it can also result from cerebral disease as well [6, 7]. MRI demonstrated no cerebellar involvement. However, the vestibulocerebellar pathway seems to be dysfunctional in the presented case.

Presence of gaze-evoked nystagmus in patients with acute vestibular syndrome is strong evidence for central involvement [8]. It is usually more severe when looking at the ipsilesional side [9]. It could be associated with spontaneous nystagmus as well, and the combination of spontaneous and gaze-evoked nystagmus could be entirely of central origin. Spontaneous nystagmus associated with gaze-evoked nystagmus is seen especially in evolving pathologies like vascular strokes. Lee and Kim have reviewed 41 patients with superior cerebellar artery infarction. They have found that gaze-evoked and spontaneous nystagmus were present in 24% of cases, and the spontaneous nystagmus in all patients was beating toward the lesion side [10]. Direction of spontaneous nystagmus is not usually conclusive for differential diagnosis. But, the presence of visual fixation is crucial to consider peripheral type of nystagmus. However, this case would probably be overlooked if she had no gaze-evoked nystagmus, and a very precious time would have been wasted before considering an urgent radiological investigation.

Choi et al. have reviewed 55 patients with combined peripheral and central disorders Central pathology was documented on MRIs. They have classified the cases into 4 groups as acute unilateral, chronic unilateral, acute bilateral, and chronic bilateral [1]. Cerebellar infarction was the most common cause for acute unilateral cases while cerebellopontine angle tumors were the most common cause in chronic unilateral cases. Wernicke encephalopathy usually presented as bilateral acute pathology and degenerative disorders were usually bilateral chronic pathologies. Simultaneous occurrence of central and labyrinthine pathologies is very rare, and several pathologies may account for this combination. Vascular cause is the leading group. The incidence of inflammatory or infectious pathology in combined cases is quite low.

There is no clear diagnosis of the combined pathology in the presented case. However, we assume that the labyrinthine involvement was secondary to intracranial process since the patient had normal hearing, and she had no story of middle ear infection. Besides, temporal bone computerized tomography showed normal ventilation of mastoid air cells. She had headache for 3 weeks, and progression of the disease to ipsilateral labyrinth would have probably accelerated her condition urging her to go to the emergency department with the symptoms of nausea and vomiting. Linear contrast enhancement of the dura throughout the tegmen indicates somehow a port of entry of the inflammatory process. However, destruction of the superior semicircular canal is quite an interesting finding since the otic capsule is one of the most solid and dense bony structure in the body. Contrast enhancement of the inflammatory mass in the labyrinth clearly indicates that the lesion is not cholesteatoma.

Isolated tumoral lesions of the inner ear, schwannoma, meningioma, lymphoma, hemangioma, metastatic tumors etc. are other possibilities for a contrast enhancing lesion which should be included in the differential diagnosis. However, they usually appear in the internal auditory canal and geniculate ganglion. Isolated semicircular canal involvement is extremely rare [11]. What type of pathology leads to lysis of the bony labyrinth in the presented case is subject to discussion. We aim to follow this patient, and the biopsy will be considered subsequently in case of hearing loss due to further labyrinthine destruction.

## Supplementary Materials

Supplementary Materials Video: the patient had mild left beating spontaneous horizontal nystagmus and slightly down-beating but intense gaze-evoked nystagmus at gaze position.
